# Silicon Nutrition Improves Lodging Resistance of Rice Under Dry Cultivation

**DOI:** 10.3390/plants14030361

**Published:** 2025-01-24

**Authors:** Hao Jiang, Zixian Jiang, Hongcheng Zhang, Yunzhe Li, Wanchun Li, Kaiyu Gao, Xintong Ma, Guan Wang, Xiaoshuang Wei, Zhihai Wu

**Affiliations:** 1 Jilin Province Green and High Quality Japonica Rice Engineering Research Center, Faculty of Agronomy, Jilin Agricultural University, Changchun 130118, China; jianghao@mails.jlau.edu.cn (H.J.); 20230789@mails.jlau.edu.cn (Z.J.); 20230806@mails.jlau.edu.cn (H.Z.); liwanchun@mails.jlau.edu.cn (W.L.); gaokaiyu0827@163.com (K.G.); maxt499@jlau.edu.cn (X.M.); wxkzbwg@163.com (G.W.); weixiaoshuang@jlau.edu.cn (X.W.); 2 Jilin Provincial Laboratory of Crop Germplasm Resources, Jilin Agricultural University, Changchun 130118, China; 3 Changchun Farmers Vocational Education Center, Changchun 130052, China; liyunzhe97@163.com

**Keywords:** rice (*Oryza sativa* L.), dry cultivation, lodging, silicon, fertilization

## Abstract

Silicon (Si) has been proven to enhance the stress resistance of rice, but its effect on the lodging resistance of rice under dry cultivation (DCR) is still unclear. The purpose of this experiment is to clarify the appropriate amount of silicon fertilizer for DCR to resist lodging and to elucidate how it coordinates lodging resistance and yield. This experiment used the ‘Suigeng 18’ cultivar as the material and set six silicon fertilizers (SiO_2_) with dosages of 0, 15, 30, 45, 60, and 75 kg·ha^−1^ (Si0, Si1, Si2, Si3, Si4, Si5). Analyze the effects and key indicators of silicon on lodging resistance of DCR from the perspectives of plant weight distribution, stem structure and composition, and root architecture. The results showed that the Si3 treatment had the highest yield and the lowest lodging index (LI). Si3 increases the weight of the upper three leaves and 4–5 internodes, thereby promoting panicle weight and yield. An increase of 13.38% in 2/3PWSI (weight of the 4th–5th stems and upper three leaves/weight of the 1st–3rd stems and lower leaves) can reflect the promoting effect of silicon on stem and leaf development near the panicle. Si3 reduces the GA/IAA value, shortens the length of the second internode, and increases the diameters of the major and minor axes, thereby increasing culm thickness and section modulus (SM), achieving the effect of “short and thick”. Si3 also increases the content of silicon and non-structural carbohydrates (NSCs) in the second internode, and increases lignin and cellulose content by upregulating the expression levels of *CAD7*, *PAL*, *COMT*, and *CesA4* genes, thereby increasing fullness and flexural strength (M), achieving “short, thick, and strong” and reducing LI. The 38.95% reduction in IFL (second internode length/fullness) reflects the positive effect of silicon on the “short, thick, and strong” stem. In the underground part, adding silicon reduces the CTK/IAA value of roots, and increases root length, root tip number, root surface area, and root weight. The key to coordinating the lodging resistance and yield of DCR with appropriate silicon dosage is to reduce the IFL in the second internode and increase 2/3 PWSI and root growth. The key to DCR and breeding is to focus on the relationship between basal internode length and fullness, as well as stem and leaf growth near the panicle.

## 1. Introduction

The International Food Policy Research Institute predicts that by 2050, the available freshwater resources are expected to decrease by 50% [1], and climate change will cause a 20% decrease in irrigated rice production in developing countries, thereby threatening world food security [2]. Dry cultivation of rice (DCR) has the characteristics of conserving water resources, simplifying production processes, and being suitable for mechanization. It is one of the important ways to address the contradiction between rice and water [3]. DCR refers to the use of live broadcasting to sow rice in dry land without going through seedling and transplanting processes. It is a rice cultivation method that mainly relies on natural precipitation throughout the entire growth period and only supplements water appropriately during critical water demand periods [4]. In recent years, progress has been made in DCR technology for increasing production and improving quality, as well as variety screening [5,6,7]. In northern China, there are often strong winds in August and September. For example, Jilin Province has been affected by typhoons such as “In-Fa”, “Bavi”, and “Maysak” since 2020, causing some crops to fall and affecting yield, increasing harvest costs. The occurrence of lodging in DCR during the grouting period will reduce production by 3.89–15.27% [8]. Therefore, lodging resistance has become one of the limiting factors for the development of DCR.

Lodging can be divided into stem lodging and root lodging, with stem lodging in the second internode being more common in China [9]. The lodging index (LI) is commonly used to characterize the lodging resistance of rice [10]. An increase in yield is often accompanied by an increase in plant height, biomass, and center of gravity height, making it prone to lodging [11,12]. However, the lodging resistance of plants depends not only on their height but also on the hardness of their stems [13]. Overall, the basal internodes of plants provide support, therefore the coordination between the hardness of the lower internodes and the weight of the upper internodes determines lodging resistance [14]. Improving the fullness of stems and leaf sheaths is an important way to increase stem strength [15]. In addition, non-structural carbohydrates (NSCs, including starch and soluble sugars) and structural carbohydrates (SCs, including cellulose and lignin) in the cell wall also determine the strength of the stem [16]. Overall, when applying agronomic measures, crop yield and lodging resistance should be considered simultaneously. However, there are currently few reports on evaluation indicators that balance DCR yield and lodging resistance.

Silicon (Si) is the second most abundant element on the surface of the Earth’s crust and in soil and has been proven to enhance crop stress resistance, especially in cereal crops such as rice [17,18,19,20]. The stress tolerance mediated by silicon is believed to be generated by two main processes, namely physical and mechanical protection, and metabolic changes induced by biochemical reactions [21,22]. Research has shown that the accumulation of silicon in plants increases the strength of cell walls, enhances the mechanical strength of rice stems, and thus improves the lodging resistance of rice [23,24]. However, under dry farming conditions, the growth cycle and root development of rice were altered, and the growth of various parts of the plant was significantly different from that of rice [4]. So, further research is needed on how silicon affects the lodging resistance of DCR, especially the impact of silicon on morphological structure, material distribution, and stem composition.

At present, there is relatively little research on the lodging resistance of DCR, especially the coordinating effect of silicon on the lodging resistance and yield of DCR, which is still unclear. Therefore, we propose two scientific questions. (1) What is the amount of silicon fertilizer that can balance yield and lodging resistance in DCR? (2) How does silicon regulate hormones and stem components to coordinate plant length, weight, and fullness, achieving a balance between yield and lodging resistance? This study aims to elucidate the mechanism and key evaluation indicators of silicon-regulated DCR lodging resistance, providing technical and theoretical support for practical production and breeding.

## 2. Results

### 2.1. Grain Yield and Lodging Index

**Figure 1:**
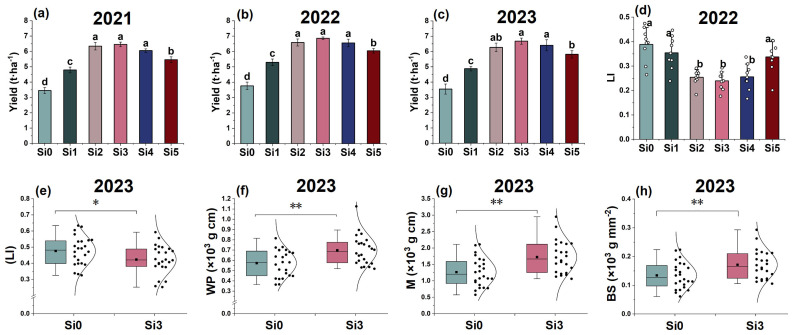
The effect of silicon nutrition on the yield and lodging rate of rice under dry cultivation. (**a**) Grain yield in 2021; (**b**) grain yield in 2022; (**c**) grain yield in 2023; (**d**) lodging index (LI) in 2022; (**e**) LI in 2023; (**f**) bending moment (WP) in 2023; (**g**) break bending moment (M) in 2023; (**h**) bending stress (BS) in 2023. Different lowercase letters indicate significant differences between different treatments (*p* < 0.05). * *p* < 0.05, ** *p* < 0.01.

We found in field trials in 2021 that adding silicon can increase DCR yield (Figure 1a). Then, the changes in production and LI were further investigated in 2022 and 2023. The yield showed a trend of first increasing and then decreasing with the increase of silicon application rate, reaching the highest level in Si3 treatment, and there was no significant difference between Si2, Si3, and Si4. The results of the three-year experiment were consistent (Figure 1a–c). The LI of Si3 is significantly lower than that of Si0, and the trend is consistent in 2022 and 2023 (Figure 1d,e). Compared with Si0, Si3 treatment significantly increased WP (Bending moment), M (Break bending moment), and BS (Bending stress) (Figure 1f–h). In summary, Si3 treatment resulted in the highest DCR yield and the lowest LI.

### 2.2. Internode Length and Center of Gravity Height

**Figure 2:**
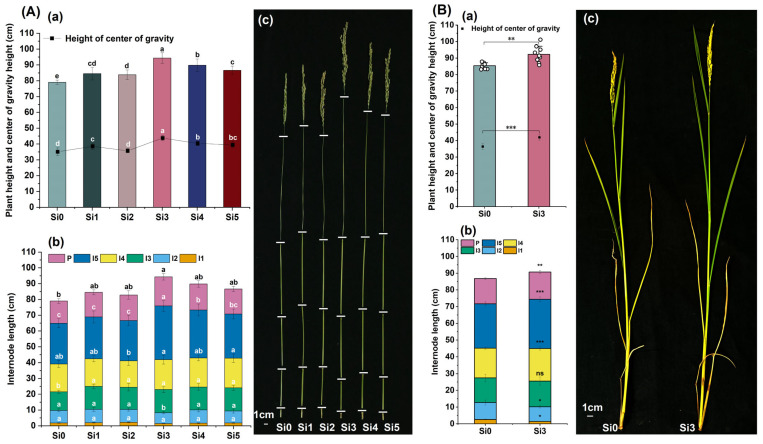
Effect of silicon nutrition on internode length and center of gravity height of rice under dry cultivation. (**A**,**B**) Plant height and center of gravity height in 2022 and 2023. (**a**) Plant height and center of gravity height; (**b**) internode length; (**c**) plant photos. Different lowercase letters indicate significant differences between different treatments (*p* < 0.05). * *p* < 0.05, ** *p* < 0.01, *** *p* < 0.001, ns, not significant.

The plant height and center of gravity height were highest and significantly higher in the Si3 treatment than in the Si0 treatment. Compared with Si0, the plant height of Si3 treatment increased by 19.38% and 8.04%, respectively, in two years, and the center of gravity height increased by 24.62% and 15.26%, respectively (Figure 2A(a) and Figure 2B(a)). The length of the first and second internodes was lower in Si3 treatment than in Si0 treatment, and the length of the second internodes showed a significant decrease over two years. There was no significant difference in the length of the first and second internodes between other treatments. Adding silicon increased the length of the third internode, but there was no significant difference in the amount of silicon applied. Among them, Si3 and Si0 treatments showed significant differences in 2022, but no significant differences were observed in 2023. The length of the fourth and fifth internodes was highest in the Si3 treatment, and compared with the Si0 treatment, the length of the fifth internodes showed significant differences in both years (Figure 2A(b,c),B(b,c)). In summary, adding silicon increased the height of the center of gravity and plant height, while Si3 treatment reduced the length of the first and second internodes and increased the length of the fourth and fifth internodes. In the two-year experiment, Si3 treatment significantly reduced the length of the second internodes and increased the length of the fifth internodes.

### 2.3. Material Accumulation and Distribution

**Figure 3:**
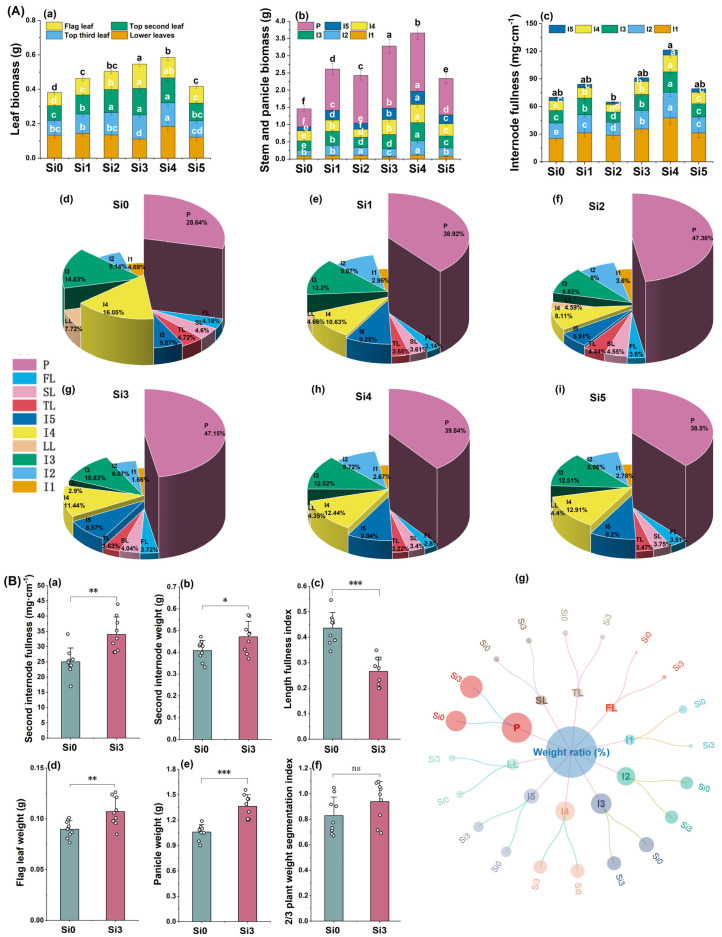
The effect of silicon nutrition on material accumulation and distribution of rice under dry cultivation. (**A**,**B**) Material accumulation and distribution in 2022 and 2023. (**A**) (**a**) Leaf biomass; (**b**) stem and panicle biomass; (**c**) internode fullness; (**d**–**i**) proportion of biomass. (**B**) (**a**) Second internode fullness; (**b**) second internode weight; (**c**) second internode length fullness index (LFI); (**d**) flag leaf weight; (**e**) panicle weight; (**f**) 2/3 plant weight segmentation index (2/3PWSI); (**g**) proportion of biomass. I1, first internode; I2, second internode; I3, third internode; I4, fourth internode; I5, fifth internode; P, panicle; FL, flag leaf; SL, top second leaf; TL, top third leaf; LL, lower leaves. Different lowercase letters indicate significant differences between different treatments (*p* < 0.05). * *p* < 0.05, ** *p* < 0.01, *** *p* < 0.001, ns, not significant.

The 2022 experiment showed that the total weight of leaves treated with Si3 and Si4 was relatively high. The weight of the upper three leaves was higher in Si2, Si3, and Si4 treatments, and there was no significant difference among the three treatments. The weight of flag leaves and second leaves was higher in Si3 and Si4 treatments. The flag leaf weight was highest and significantly higher in Si3 treatment than in other treatments, and lowest in Si0 treatment. Among the Si1-Si5 treatments, Si3 had the highest proportion of flag leaves (3.72%) and the lowest proportion of lower leaves (2.90%) (Figure 3A(a,d-i)). In terms of stem weight. The weight of the first and second internodes in the Si3 treatment was smaller and significantly lower than that in the Si0 treatment. The proportion of weight between the first and second internodes in Si3 treatment was the smallest, at 1.66% and 6.07%, respectively. The fullness of the first and second internodes was significantly higher than that in the Si0 treatment and only lower than that in the Si4 treatment (Figure 3A(b,c,g)). The weight and fullness of the third, fourth, and fifth internodes were highest in the Si4 treatment, followed by the Si3 and Si1 treatment (Figure 3A(c)). In terms of panicle weight, the panicle weight was highest and significantly higher in Si3 treatment than in other treatments, accounting for 47.15% of the panicle weight (Figure 3A(b,g)). From the overall perspective of the plant, Si3 treatment had the lowest weight proportion of the lower part (1st to 3rd stems and the lower leaves) at 21.46%, while the highest weight proportion of the upper part (4th to 5th stems, upper three leaves and panicle) was 78.54% (Figure 3A(g)). In summary, the 2022 experimental results indicate that Si3 and Si4 treatments have advantages in terms of upper leaf weight, basal internode fullness, and panicle weight of plants. Si3 treatment reduces the proportion of lower leaf weight and increases flag leaf weight and panicle weight while maintaining high fifth internode weight and fullness.

The 2023 experiment is consistent with the trend of 2022. Compared with Si0 treatment, Si3 treatment significantly improved the fullness and weight of the second internode, while significantly reducing the LFI (Length fullness index) of the second internode by 38.95% (Figure 3B(a–c)). Si3 treatment significantly increased flag leaf weight and panicle weight, and increased their proportion (Figure 3B(d,e)). The proportion of lower plant weight in Si0 and Si3 treatments was 38.48% and 33.48%, respectively, and the proportion of upper plant weight was 61.52% and 66.52%, respectively (Figure 3B(g)). The 2/3 PWSI (2/3 plant weight segmentation index) of the Si3 treatment is 13.38% higher than that of the Si0 treatment (Figure 3B(f)). In summary, adding silicon increases the fullness of the second internode, promotes the growth of the flag leaf, fifth internode, and spike, while ensuring lodging resistance, and thus increases the proportion of the upper part of the plant. The decrease in LFI and the increase in 2/3 PWSI can well reflect the role of silicon addition in improving lodging resistance and yield.

### 2.4. Cross-Sectional Structure of Stem and Plant Hormone Content

**Figure 4:**
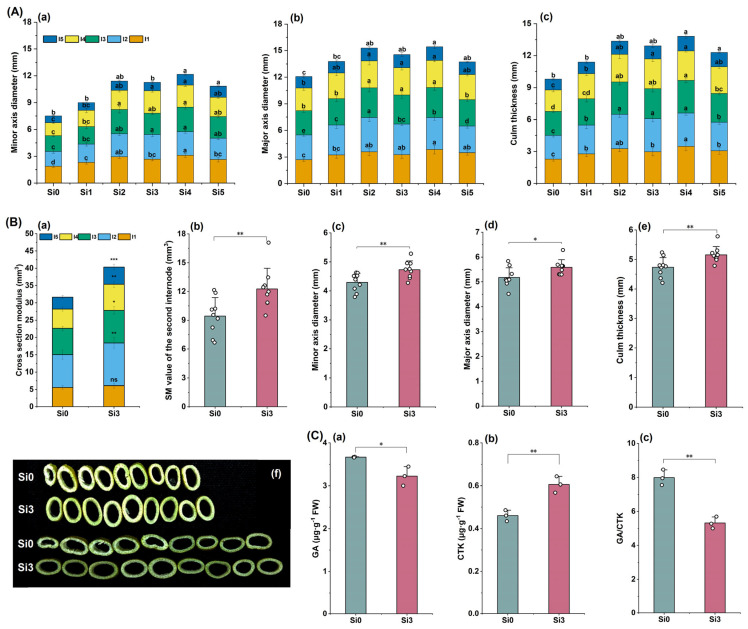
The effect of silicon nutrition on the stem cross-sectional structure and plant hormone content of rice under dry cultivation. (**A**,**B**) Stem cross-sectional structure and hormone content in 2022 and 2023. (**A**) (**a**) Minor axis diameter; (**b**) major axis diameter; (**c**) culm thickness. (**B**) (**a**) Cross-section modulus (SM); (**b**) SM value of the second internode; (**c**) minor axis diameter; (**d**) major axis diameter; (**e**) culm thickness; (**f**) cross-sectional photo of the second internode. (**C**) (**a**) Gibberellin (GA) content in the second internode; (**b**) content of cytokinin (CTK) in the second internode; (**c**) second internode GA/CTK. Different lowercase letters indicate significant differences between different treatments (*p* < 0.05). * *p* < 0.05, ** *p* < 0.01, *** *p* < 0.001, ns, not significant.

The experimental results in 2022 showed that the minor axis diameter and major axis diameter of each internode were higher in Si2, Si3, and Si4 treatments, and were significantly higher than those in Si0 treatment (Figure 4A(a,b)). The culm thickness of the second internode was higher in Si2, Si3, and Si4 treatments, and there was no significant difference among the three treatments (Figure 4A(c)). The 2023 experiment further showed that the cross-section modulus (SM) of each internode in the Si3 treatment was higher than that in the Si0 treatment, and the second internode reached a significant level (Figure 4B(a,b)). Si3 treatment significantly increased the minor axis diameter, major axis diameter, and stem diameter of the second internode (Figure 4B(c–f)). Compared with Si0, the GA content in the second internode of Si3 treatment was significantly reduced, the CTK content was significantly increased, and the GA/CTK ratio was significantly reduced (Figure 4C(a–c)). In summary, adding silicon promotes lateral development of the second internode and increases the culm thickness and SM.

### 2.5. Carbohydrate and Silicon Content and Gene Expression Levels in the Second Internode

**Figure 5:**
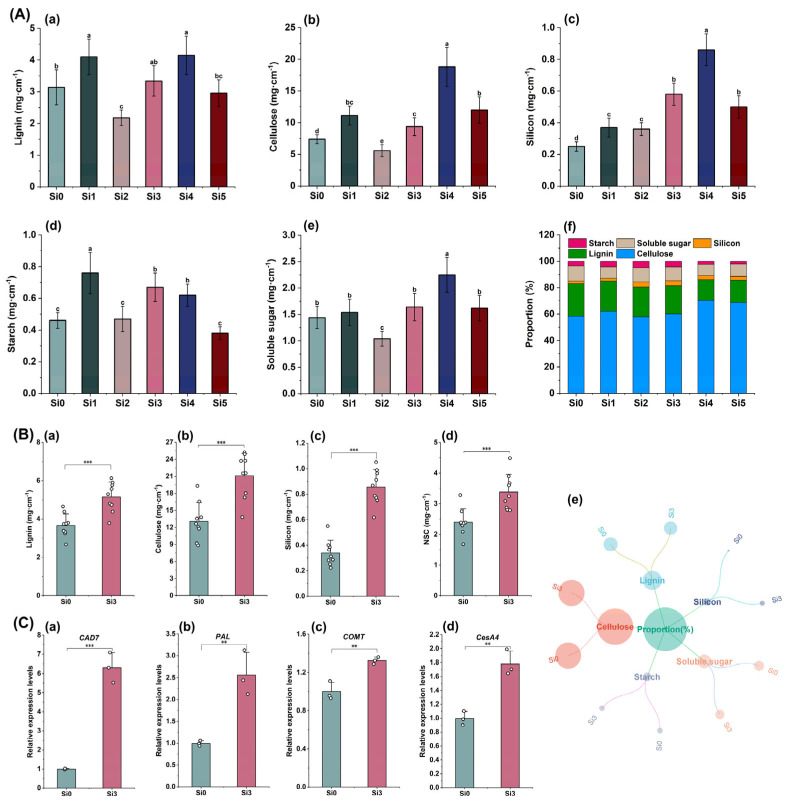
The effect of silicon nutrition on carbohydrate and silicon content and gene expression in the second internode of rice under dry cultivation. (**A**) Research results for 2022; (**B**,**C**) research results for 2023. (**A**) (**a**) Lignin content; (**b**) cellulose content; (**c**) silicon content; (**d**) starch content; (**e**) soluble sugar content; (**f**) proportion of each component. (**B**) (**a**) Lignin content; (**b**) cellulose content; (**c**) silicon content; (d) non-structural carbohydrate (NSC) content; (**e**) proportion of each component. (**C**) Relative expression level of genes. (**C**) (**a**) *CAD7*; (**b**) *PAL*; (**c**) *COMT*; (**d**) *CesA4*. Different lowercase letters indicate significant differences between different treatments (*p* < 0.05). ** *p* < 0.01, *** *p* < 0.001.

The 2023 experiment showed that the lignin content was higher in Si4 treatment, followed by Si1 and Si3 treatment (Figure 5A(a)). The cellulose content was significantly higher in all treatments except for the Si2 treatment compared to the Si0 treatment (Figure 5A(b)). The silicon content was highest in Si4 treatment, followed by Si3 and Si5 treatment, and there was no significant difference between Si3 and Si5 (Figure 5A(c)). There was no significant difference in starch content between the Si3 and Si4 treatments, but both were significantly higher than the Si0 treatment (Figure 5A(d)). Soluble sugars were highest in the Si4 treatment, followed by the Si3 treatment (Figure 5A(e)). The proportion of each component showed that Si4 treatment had the highest proportion of cellulose reaching 70.45%, while lignin and soluble sugar had the lowest proportion. The overall proportion of starch and soluble sugars was higher in Si2 and Si3 treatments, at 15.72% and 14.79%, respectively. The proportion of silicon content is also higher in Si2 and Si3 treatments, at 3.76% and 3.72%, respectively (Figure 5A(f)). The 2023 experiment showed that, compared with Si0, Si3 significantly increased the content of lignin, cellulose, silicon, and non-structural carbohydrates (NSCs) and increased the proportion of cellulose (Figure 5B(a–e)). Si3 treatment significantly upregulated the expression levels of *CAD7*, *PAL*, *COMT*, and *CesA4* (Figure 5C(a–d)). In summary, Si3 treatment increased silicon accumulation in the second internode, upregulated the expression of structural carbohydrate-related genes, and increased the content of non-structural carbohydrates.

### 2.6. Root Architecture and Hormone Content

**Figure 6:**
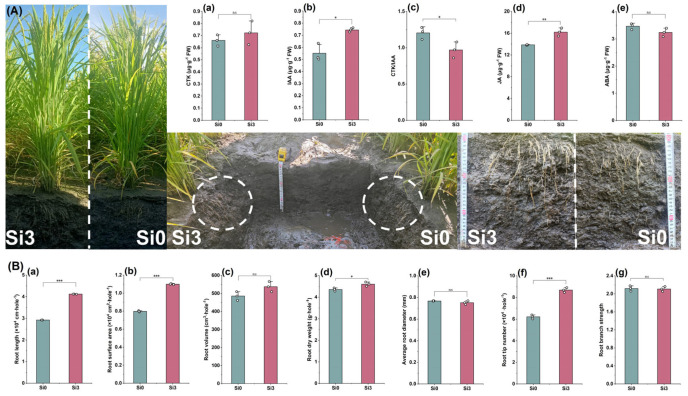
The effect of silicon nutrition on root architecture and hormone content of rice under dry cultivation. (**A**) (**a**) CTK content; (**b**) auxin (IAA) content; (**c**) CTK/IAA; (**d**) jasmine acid (JA) content; (**e**) abscisic acid (ABA) content. (**B**) (**a**) Root length; (**b**) root surface area; (**c**) root volume; (**d**) root dry weight; (**e**) average root diameter; (**f**) root tip number; (**g**) root branch strength. * *p* < 0.05, ** *p* < 0.01, *** *p* < 0.001.

The research results in 2023 showed that Si3 increased the CTK content in roots but did not reach a significant level. Si3 significantly increased the IAA and JA content in roots, while significantly reducing the CTK/IAA content (Figure 6A(a–d)). In addition, Si3 reduced the root ABA content, but did not reach a significant level (Figure 6A(e)). In terms of root system configuration, Si3 treatment significantly increased root length, root surface area, and root dry weight, while increasing root volume but not reaching a significant level (Figure 6B(a–d)). Si3 treatment had no significant effect on the average root diameter and branch strength, but significantly increased the number of root tips (Figure 6B(e–g)). In summary, adding silicon reduced the CTK/IAA value and JA content of DCR, promoted root growth, and increased the contact area between roots and soil.

## 3. Discussion

### 3.1. The Effect of Silicon on the Yield and Lodging Index of Rice Under Dry Cultivation

**Figure A1:**
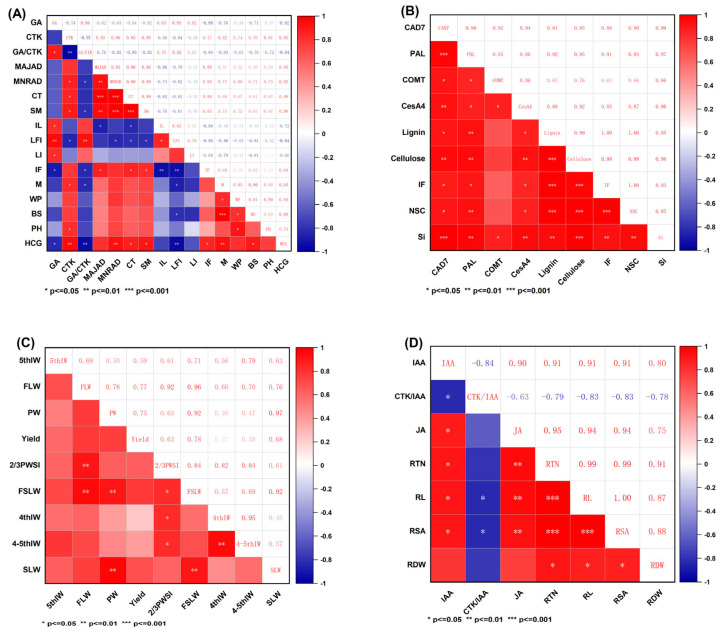
(**A**–**D**). Correlation analysis. MAJAD, major axis diameter; MNRAD, minor axis diameter; CT, culm thickness; SM, cross-section modulus; IL, internode length; LFI, second internode length fullness index; LI, lodging index; IF, internode fullness; M, break bending moment; WP, bending moment; BS, bending stress; PH, plant height; HCG, height of center gravity; NSC, non-structural carbohydrates; 5thIW, 5th internode weight; 4thIW, 4th internode weight; 4–5thIW, the weight of the fourth to fifth internodes; FLW, flag leaf weight; SLW, second leaf weight; FSLW, weight of the upper two leaves; 2/3PWSI, 2/3 plant weight segmentation index; PW, panicle weight; RTN, root tip number; RL, root length; RSA, root surface area; RDW, root dry weight.

With the increase in extreme weather, researchers are constantly exploring ways to coordinate yield and lodging resistance. Usually, yield increase is achieved by increasing planting density and fertilization. However, higher populations lead to internode elongation, significant reduction in stem diameter and wall thickness, and easy breakage or bending of stems, thereby increasing the risk of lodging [25,26]. LI is determined from two metrics, M and WP. When WP is small and M is large, the anti-lodging ability is enhanced. When too much fertilizer is applied, the M value will decrease, thereby increasing the risk of lodging [14]. In this experiment, Si3 treatment increased yield while reducing Li. Although high yield was inevitably accompanied by an increase in WP, silicon addition mainly reduced LI by increasing stem M value, especially significantly increasing the second internode SM. BS was determined by the ratio of M to SM, and excessive use of fertilizer can lead to a decrease in stem SM and an increase in BS [15]. Compared with Si0, Si3 treatment increased SM and M in the second internode, thereby improving BS, and M was significantly positively correlated with BS (Figure A1A). In summary, this experiment found that adding silicon increased the SM of each internode, especially significantly promoting the SM and M values of the second internode (Figure 4B(a,b)), which was the main reason for reducing the lodging index. When considering both yield and lodging resistance, in addition to stem bending resistance, attention should also be paid to the length, weight, and distribution characteristics of various parts of the plant. So, how does adding silicon affect the length, weight, and distribution of various parts of DCR?

### 3.2. The Effect of Silicon Nutrition on Stem and Leaf Growth and Weight Distribution of Rice Under Dry Cultivation

Experiments have shown that increasing plant height and center of gravity height can increase the risk of lodging and lead to yield loss [26,27]. Some researchers have also found that plant lodging depends on stem strength [13]. We found that the Si3 treatment with the highest yield and lowest LI had the highest plant height and center of gravity height, mainly due to the increase in panicle weight, which in turn increased the center of gravity height. The growth of lower internodes in cereal crops can lead to a decrease in M, which in turn makes the crops prone to lodging [28]. We found that Si3 significantly reduces the length of the second internode and has higher fullness, which is the reason for improving the ability to resist lodging. In addition, increasing the length of the basal internodes of the stem can sometimes be accompanied by a decrease in the length of the upper internodes, altering plant structure and reducing stress resistance [29]. We found that Si3 treatment reduced the length of the first and second internodes and increased the length of the fourth and fifth internodes, while increasing the fullness of the first and second internodes. The fullness of the second internodes was positively correlated with SM (Figure A1A). The first and second internodes of Si4 treatment have higher fullness, but the basal internodes are longer than those of Si3 treatment. The proportion of lower leaves is higher and the flag leaf weight is smaller, so there is no advantage in lodging resistance and yield. It can be seen that the length and fullness of internodes should be important considerations for evaluating lodging resistance. So, we used the length of the second internode divided by the degree of fullness to form an index and named it the length fullness index (LFI). We found that LFI is positively correlated with Li (Figure A1B). The shorter and greater the fullness of the second internode, the stronger its ability to resist lodging. So, how does adding silicon affect the length and fullness of the second node?

The SCs and NSCs in the stem represent the stability and stress resistance of the stem [30,31,32]. The accumulation of silicon in stems is an important factor in improving lodging resistance [24]. This experiment found that adding silicon promoted the content of carbohydrates and Si in the second internode. The plumpness of the second internode is positively correlated with the content of NSCs, lignin, cellulose, and Si (Figure A1B). Research has shown that cinnamyl alcohol dehydrogenase (CAD), phenylalanine transaminase (PAL), and caffeic acid-O-methyltransferase (COMT) are key enzymes in lignin synthesis [33]. On the other hand, cellulose synthase (CesA) is a key enzyme in cellulose synthesis [34]. This experiment found that adding silicon upregulated the expression levels of lignin synthesis-related genes *CAD7*, *PAL*, *COMT*, and cellulose synthesis gene *CesA4*. In addition, this experiment showed a significant positive correlation between GA and internode length, and silicon addition reduced internode length by lowering GA and GA/CTK in the second internode. Adding silicon increases the content of CTK in the second internode and promotes the minor axis and major axis diameters. The minor axis diameter and culm thickness of the second internode are positively correlated with CTK and negatively correlated with GA/CTK (Figure A1A). In summary, adding silicon increases the second internode fullness by promoting carbohydrate and Si accumulation, shortens the second internode length, and increases culm thickness by reducing GA/CTK values, which is the reason for increasing the SM and fullness of the second internode and reducing the LFI.

This experiment found that the effect of Si on DCR yield should consider both leaf biomass and its proportion in the plant. For example, the Si0 treatment had a higher proportion of flag leaves due to stem development obstruction, but the flag leaf weight was the lowest, and the proportion of lower leaves was the highest among the Si0–Si5 treatments. The Si3 treatment shortens the first and second internodes and increases fullness. Its lower leaf proportion is the lowest among the Si0–Si5 treatments, and it also has the highest flag leaf weight. Research has found that flag leaves contribute significantly to panicle weight [8]. This experiment found a positive correlation between the weight of the upper two leaves and panicle weight, and the addition of silicon promoted the weight of the upper fourth and fifth nodes and the upper two leaves of the plant. So, we seem to have discovered the secret of Si balancing yield and resistance to lodging. That is to promote the growth of flag leaves and the fifth internode, while reducing the LFI of the second internode, to ensure the lower part’s lodging resistance and increase the proportion of the upper part of the plant. We use the weight of the upper part of the plant (the weight of the 4th to 5th stems and the weight of the upper three leaves) divided by the weight of the lower part (the weight of the 1st to 3rd stems and the lower leaves) to form an index, named the 2/3 plant weight segmentation index (2/3PWSI), to analyze the proportional relationship between the upper and lower parts of the plant after silicon addition. We found that adding silicon increased 2/3 PWSI, and 2/3 PWSI was positively correlated with panicle weight (Figure A1C). In summary, adding silicon ensures lodging resistance by reducing the second internode LFI and promotes panicle growth by increasing plant 2/3 PWSI, thereby balancing lodging resistance and yield.

### 3.3. The Effect of Silicon on Root Architecture and Plant Hormones of Rice Under Dry Cultivation

A higher root weight and a flat root shape are beneficial for material transport in plant stems and enhance lodging resistance [35]. Therefore, it is necessary to combine root morphology with stem characteristics to explore lodging resistance. This experiment found that the root distribution of Si3 treatment was wider (Figure 6A), which may have increased the contact area between the roots and the soil, providing a better grip for the plants. Further research has shown that the addition of silicon significantly increases root length, surface area, root weight, and root tip number, while there is no significant difference in branch strength and root diameter. This suggests that silicon may increase root surface area and root weight by promoting fine root length and root tip number. Analysis shows that the number of root tips, root length, root surface area, and root weight are positively correlated (Figure A1D). The balanced relationship between IAA and CTK signals affects the growth pattern of roots [36,37]. Research has shown that when callus tissue accumulates high concentrations of IAA, it promotes root differentiation, while when CTK accumulates more, it activates the expression of bud genes, thereby promoting bud growth [38]. This experiment found that Si3 treatment significantly increased the IAA content in roots, thereby reducing the CTK/IAA value. Further analysis showed that CTK/IAA was negatively correlated with root length and root surface area. Silicon addition significantly increased the JA content in the root system, and JA and IAA were positively correlated with root length, root surface area, and root tip number. ABA showed no significant changes. In summary, adding silicon promotes the content of IAA and JA in the roots of rice under dry cultivation and promotes root growth, which is beneficial for improving lodging resistance and material accumulation.

## 4. Materials and Methods

### 4.1. Site Description

Completed field experiments at Jilin Agricultural University (125°40′ E, 43°81′ N) from 2021 to 2023. In 2021, soil nutrients were measured before sowing. The pH value of the soil at a depth of 0–30 cm in the experimental field is 6.5, the organic matter is 19.85 g·kg^−1^, the available silicon is 86.56 mg·kg^−1^, the available nitrogen is 95.70 mg·kg^−1^, the available phosphorus is 26.63 mg·kg^−1^, and the available potassium is 156.35 mg·kg^−1^. This area has a humid continental climate and experienced average temperatures of 18.45 °C, 18.34 °C, and 18.96 °C over the three-year period, along with annual precipitation levels of 714.8 mm, 697.7 mm, and 465.8 mm, respectively.

### 4.2. Experimental Design and Crop Management

This study used ‘Suigeng 18’ (China Rice Data Center, No. 2014021, https://www.ricedata.cn/variety/varis/614593.htm, accessed on 1 January 2020) as the experimental material. The rice variety ‘Suigeng 18’ was selected because it is a variety provided for dry farming in the central region of Jilin Province [7,8].

Field experiments were conducted in 2021, 2022, and 2023. Six levels of silicon fertilizer (SiO_2_) were set up for the experiment, namely 0 kg·ha^−1^ (Si0), 15 kg·ha^−1^ (Si1), 30 kg·ha^−1^ (Si2), 45 kg·ha^−1^ (Si3), 60 kg·ha^−1^ (Si4) and 75 kg·ha^−1^ (Si5). The experimental plot covers an area of 18 m^2^ and is sown using dry live broadcasting with a seeding rate of 195 kg·ha^−1^. The plot is conducted using strip sowing with a row spacing of 30 cm, and each treatment is repeated three times. In 2021, the actual yield of six treatments was measured, and in 2022, the lodging index, stem morphology, plant biomass, and stem composition of the six treatments were measured, and suitable amounts of silicon fertilizer were found. In 2023, the differences between the two treatments of no silicon (Si) and the most suitable silicon (Si3) were analyzed, and the mechanism by which the appropriate amount of silicon applied increases lodging resistance was elucidated.

Simultaneously apply silicon fertilizer, phosphorus fertilizer, and potassium fertilizer as base fertilizers and apply them all at once. Silicon fertilizer is made of BioPower Russian mineral silicon (SiO_2_ content ≥ 72%). The use of superphosphate (P_2_O_5_ 12%) for phosphorus fertilizer and potassium chloride (K_2_O 60%) for potassium fertilizer is both 75 kg·ha^−1^. Nitrogen fertilizer uses urea (pure N 46%) at a rate of 160 kg·ha^−1^. Nitrogen fertilizer is applied in a ratio of 5:3:2 during the soil preparation stage, tillering stage, and booting stage. The whole growth period is mainly rain-fed, and a soil water potential analyzer (SYS-TSS1; Sayas Technology Co., Ltd., Liaoning, China) is used to monitor the changes in water potential in the experimental field. When the soil water potential of 10–15 cm is below −35 kPa, a fixed spray 360° atomized rotary sprinkler is used to replenish water until the soil water potential reaches −10 kPa. Sowing is completed in early May and harvesting is completed in early October every year. After sowing and before emergence, bensulfuron-methyl combined with butralin was sprayed for soil sealing. When the rice seedlings were at the two-leaf and one-heart stages, cyhalofop-butyl was sprayed to control grassy weeds. Around the three-leaf stage, bentazone was sprayed according to the situation of broadleaf weeds. No other growth regulators were used. Other measures are managed according to the requirements of high-yield cultivation.

### 4.3. Sampling and Measurements

#### 4.3.1. Yield

During the mature stage, take 1 m^2^ of rice panicles from the field to measure their weight (kg) and calculate the actual yield, repeating the process three times.

(1) $$Yield\;(t\cdot {ha}^{-1})=panicle\;weight\;per\;m^{2}\;(kg)\times 10000/1000.$$

#### 4.3.2. Mechanical and Morphological Characteristics of Stem

##### Mechanical Characteristics of Stem

During the flowering stage, 60 main stems with the same flowering period and consistent plant height were selected and labeled in each community. During the grouting stage, 24 representative plants were selected for each treatment. The flexural strength was measured using a stem strength tester (YYD-1; Zhejiang Top Instrument Co., Ltd., Hangzhou, China). The length (SL) and fresh weight (FW) from the base of the second internode to the top of the panicle were measured. The calculation method is as follows [14].

(2) $$\begin{array}{cc} Bending\;moment & \left(WP,g\cdot cm\right)\\ & =Length\;from\;broken\;point\;to\;spike\;top\;(SL,cm)\\ & \times \;Fresh\;weight\;of\;the\;plant\;material\;above\;the\;broken\;point\;(FW,g). \end{array}$$

(3) $$\begin{array}{cc} Break\;bending & moment\;(M,g\cdot cm)\\ & \;=\;1/4\;\times \;Flexural\;strength\;(BL,kg)\\ & \;\times \;Distance\;between\;two\;support\;points\;(L,cm)\times 10^{3} \end{array}$$

(4) Lodging index (LI)=WP/M.

##### Stem Morphological Characteristics

During the grouting stage, 9 representative plants were selected for each treatment, and their plant height and center of gravity height were measured. There following steps were then carried out: Decompose the plant leaves into flag leaves (FL), top second leaves (SL), top third leaves (TL), and lower leaves (LL). Starting from the base of the stem, measure the length of each internode and panicle in sequence (I1, I2, I3, I4, I5, P). Measure the major axis diameter (b1, mm), major axis inner diameter (b2, mm), minor axis diameter (a1, mm), and minor axis inner diameter (a2, mm) of each internode. Finally, bag the leaves of each part and the culms, sheaths, and panicles of each internode separately. Use an oven to kill them at 105 °C for 30 min, then dry them at 80 °C to a constant weight and weigh them. The calculation method is as follows [16].

(5) $$\begin{array}{cc} Section\;modulus & \left(SM,mm^{3}\right)\\ & =\pi /32\times (minor\;axis\;diameter\;{a1}^{3}\times major\;axis\;diameter\;b1\\ & -\;minor\;axis\;inner\;diameter\;{a2}^{3}\\ & \times \;major\;axis\;inner\;diameter\;b2)/al. \end{array}$$

(6) $$Bending\;stress\;(BS,g\cdot mm^{-2})=M\times 10/SM.$$

(7) $$\begin{array}{cc} Internode & fullness\;(IF,mg\cdot {cm}^{-1})\\ & \;\;\;=\;Culm\;dry\;weight\;(mg)/internode\;length(cm). \end{array}$$

(8) Culm thickness (mm)=(a1+b1)/2.

(9) Length fullness index (LFI) = Second internode length/internode fullness.

(10) 2/3 plant weight segmentation index (2/3PWSI)=The weight of the 4th to 5th stems and the weight of the upper three leaves/the weight of the 1st to 3rd stems and the lower leaves.

#### 4.3.3. Stem Carbohydrates and Silicon Content

##### Carbohydrate Content

Grind the dried stem sample and pass it through a 60-mesh sieve. Determine soluble sugar and starch content using an improved sulfuric acid anthrone colorimetric method [39]. NSCs are the sum of starch and soluble sugar content. Determine cellulose content according to Zhang et al.’s method [40]. Firstly, add 5 mL of acetic acid/nitric acid reagent to 0.1 g of powder sample and boil for 30 min. After centrifugation, pour out the supernatant and rinse the precipitate three times with 5 mL of distilled water. Then, add 2.5 mL of 72% sulfuric acid and incubate at room temperature for 12 h. Add 5 mL of distilled water to each test tube. After rinsing the test tube three times, combine all washings and hydrolyze cellulose contents into a 20 mL volumetric flask. Finally, measure the hydrolyzed cellulose as glucose equivalent using the aforementioned anthrone method.

Use Ishimaru et al.’s method to determine lignin content [41]. Extract the sample with ethanol ether (*v*/*v* = 1:1) and dry it. Then, add 10 mL of 72% sulfuric acid to 0.5 g of dry sample and incubate at room temperature for 8 h. Then, pour the mixture into 280 mL of water and boil for 4 h, followed by filtration and acid removal. Subsequently, dry the filter and lignin to achieve a constant weight. Calculate the lignin content as follows: Cw (mg·g^−1^) = 1000 × W/S, where W is the pellet weight (g), and S is the dried sample weight (g).

##### Silicon Content

Add 0.1 g of sample into a digestion tube, then add 3 mL of nitric acid and 1 mL of hydrofluoric acid, and use a digestion instrument (MD20H; APL Instrument Co., Ltd., Jilin, China) for digestion. After completion, place the digestion tube in a graphite acid removal instrument (GD25; APL Instrument Co., Ltd., Jilin, China) for acid removal. Transfer the acid-washed sample to a 100 mL volumetric flask to a constant volume for testing. Determine the Si content using an inductively coupled plasma emission spectrometer (ICP-OES Agilent 710; Agilent Technologies, Palo Alto, CA, USA).

#### 4.3.4. Root Architecture

Take out the soil 30 cm deep next to the roots of plants in the field, observe the distribution of roots, and take photos. Use a root morphology scanner (Epson Perfection V800 photo; EPSON, Tokyo, Japan) to scan root images and store them in a computer. Use the root analysis system software WinRhizo PRO 2016 (Regent Instruments, Québec, Canada) to analyze root length, root surface area, root volume, average diameter, and root tip number. Record the weight after drying the roots. Branch strength = number of root tips/root length.

#### 4.3.5. Hormone Content

Fresh samples were taken from the second internode of the base and the root system to determine the content of gibberellin (GA) and cytokinin (CTK) in the second internode, as well as the content of CTK, indole-3-acetic acid (IAA), jasmonic acid (JA), and abscisic acid (ABA) in the roots. Then, the following steps were performed: Take 1 g of the sample, grind it to a dry powder in liquid nitrogen, and extract it with a mixture of isopropanol water hydrochloric acid. Add 8 μL lμg·mL^−1^ of internal standard solution, and shake at low temperature for 30 min. Add dichloromethane, shake at low temperature for 30 min, centrifuge at 13,000 r/min for 5 min, and take the lower organic phase. Under dark conditions, dry the organic phase with nitrogen and dissolve it in methanol. Centrifuge at 13,000 r/min for 10 min at 4 °C, and take the supernatant and filter it through a 0.22 μm membrane. Determine using ionization (ESI) high-performance liquid chromatography–tandem mass spectrometry (Agilent 1290; Agilent, Palo Alto, CA, USA. SCIEX-6500 Qtrap; Applied Biosystems SCIEX, Washington D.C., WA, USA).

#### 4.3.6. Gene Expression Level

Freeze the second internode and root system with liquid nitrogen and transfer them to a −80 °C freezer.

After grinding the tissue in liquid nitrogen, extract the total RNA using TriPure reagent (Aidlab Biotechnology). Reverse transcribe RNA into single-stranded cDNA using ReverTra Ace qPCR RT Master Mix (TOYOBO). Adopt real-time fluorescent quantitative PCR amplification using cDNA as a template. Use a 20 uL reaction system, 10 μL of 2×SuperReal PreMix Plus, 8 μL of ddH_2_O, 0.5 μL of upstream and downstream primers, and 1 μL of cDNA template. Pre-denature the PCR amplification reaction system at 95 °C for 15 min. Perform the following 40 cycles: pre-denaturation at 95 °C for 20 s, annealing at 60 °C for 20 s, extension at 72 °C for 20 s, and melting for 6 s. Use the 2^–∆∆CT^ method for relative gene expression analysis, and primer information is shown in Table A1.

#### 4.3.7. Statistical Analysis

Microsoft Excel 2021 (Microsoft, Redmond, WA, USA) was used to organize the data. All experiments were conducted in triplicate (*n* = 3). One-way analysis of variance (ANOVA) and statistical analysis was performed to determine statistically significant differences between the control and other treatments using SPSS 27 software (SPSS, Chicago, IL, USA). At *p* < 0.05, the difference was considered statistically significant. Origin 2022 software (OriginLab, Northampton, MA, USA) was used to generate figures.

## 5. Conclusions

**Figure 7:**
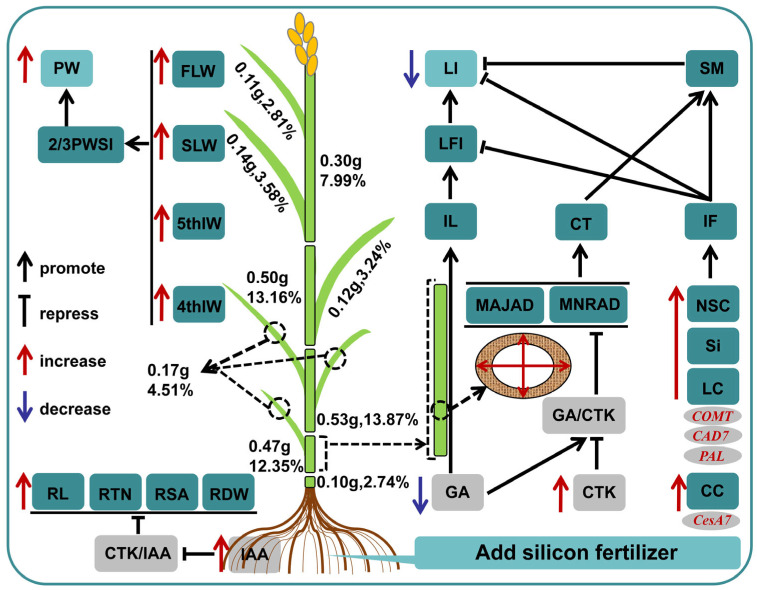
A descriptive model for enhancing the lodging resistance of rice under dry cultivation by adding silicon fertilizer. In the figure, the red font indicates activation, and the blue font indicates inhibition. Numbers and percentages represent the weight and proportion of the area. MAJAD, Major axis diameter; MNRAD, minor axis diameter; CT, culm thickness; SM, cross-section modulus; IL, internode length; LFI, second internode length fullness index; LI, lodging index; IF, internode fullness; M, break bending moment; WP, bending moment; BS, bending stress; PH, plant height; HCG, height of center gravity; NSCs, non-structural carbohydrates; LC, lignin content; CC, cellulose content; 5thIW, 5th internode weight; 4thIW, 4th internode weight; 4–5thIW, the weight of the fourth to fifth internodes; FLW, flag Leaf weight; SLW, second leaf weight; FSLW, weight of the upper two leaves; 2/3PWSI, 2/3 plant weight segmentation index; PW, panicle weight; RTN, root tip number; RL, root length; RSA, root surface area; RDW, root dry weight.

We found that adding suitable silicon fertilizer (45 kg·ha^−1^) (Si3) can improve the lodging resistance of rice under dry cultivation and we established a descriptive model (Figure 7). Si3 treatment promotes root growth, laying the foundation for improving plant grip and material accumulation. The basal internodes of plants play a crucial role in lodging resistance. Si3 shortens the length of the second internode by reducing GA/IAA and increases the culm thickness and cross-section modulus of the second internode. Si3 improves fullness and flexural strength by promoting carbohydrate accumulation, thereby reducing IFL and LI. Si3 increases the weight of the upper three leaves and the 4th–5th internodes while reducing the proportion of lower leaves, providing a material basis for the nutritional growth of the panicle, thereby increasing 2/3 PWSI and panicle weight. In summary, adding silicon coordinates lodging resistance and yield by affecting the internode length and weight distribution of various parts of the plant. IFL and 2/3PWSI can serve as important reference indicators in breeding and cultivation. These findings provide new solutions for DCR production and expand our understanding of the coordinated relationship between silicon-regulated DCR lodging resistance and yield.

## Data Availability

Data will be made available on request. The data are not publicly available due to privacy.

## Appendix A

**Table A1:** Design of RT-qPCR reaction primer.

Gene Names	Primer	Product Size
*CAD7*	F: CAAGCACATCCATGCACTCT	168 bp
	R: TCATCTCCTGCGTTTCACTG	
*PAL*	F: AGCTTGGACTACGGCTTCAA	201 bp
	R: GAGGAAGGTGGAGGACATGA	
*COMT*	F: GCCATCCTGATGAAGTGGAT	233 bp
	R: AGCTCCCTGAACTCCCTCTC	
*CesA4*	F: AGGCAAGCGCTCTATGGTTA	208 bp
	R: GCATCGAGCGTTCATACTCA	
